# Efficacy and safety profile of biotechnological agents and Janus kinase inhibitors in VEXAS syndrome: data from the international AIDA Network VEXAS registry

**DOI:** 10.3389/fphar.2025.1462254

**Published:** 2025-02-19

**Authors:** Antonio Vitale, Valeria Caggiano, Flavia Leone, Andrea Hinojosa-Azaola, Eduardo Martín-Nares, Guillermo Arturo Guaracha-Basañez, Jiram Torres-Ruiz, Perla Ayumi Kawakami-Campos, Pravin Hissaria, Alicia Callisto, Mark Beecher, Lorenzo Dagna, Corrado Campochiaro, Alessandro Tomelleri, Micol Frassi, Franco Franceschini, Francesca Crisafulli, José Hernández-Rodríguez, Verónica Gómez-Caverzaschi, Olga Araújo, Paolo Sfriso, Sara Bindoli, Chiara Baggio, Jurgen Sota, Abdurrahman Tufan, Hamit Kucuk, Matteo Piga, Alberto Cauli, Maria Antonietta D’Agostino, Amato De Paulis, Ilaria Mormile, Henrique A. Mayrink Giardini, Rafael Alves Cordeiro, Giuseppe Lopalco, Florenzo Iannone, Sara Monti, Carlomaurizio Montecucco, Guillermo Ruiz-Irastorza, Adriana Soto-Peleteiro, Paola Triggianese, Carmelo Gurnari, Ombretta Viapiana, Riccardo Bixio, Rosetta Vitetta, Guido Rovera, Edoardo Conticini, Francesco La Torre, Piero Portincasa, Nour Jaber, Gaafar Ragab, Amina Maher, Ezgi Deniz Batu, Seza Ozen, Ewa Wiesik-Szewczyk, Alejandra de-la-Torre, Alberto Balistreri, Bruno Frediani, Claudia Fabiani, Luca Cantarini

**Affiliations:** ^1^ Department of Medical Sciences, Surgery and Neurosciences, Research Center of Systemic Autoinflammatory Diseases and Behçet’s Disease Clinic, University of Siena, Siena, Italy; ^2^ Autoinflammatory and Autoimmune Diseases (RITA) Center Siena, Azienda Ospedaliero-Universitaria Senese European Reference Network (ERN) for Rare Immunodeficiency, Siena, Italy; ^3^ UOC di Reumatologia, Fondazione Policlinico Universitario A Gemelli IRCCS, Università Cattolica del Sacro Cuore, Roma, Italy; ^4^ Department of Immunology and Rheumatology, Instituto Nacional de Ciencias Médicas y Nutrición Salvador Zubirán, Mexico City, Mexico; ^5^ Department of Ophthalmology, Instituto Nacional de Ciencias Médicas y Nutrición Salvador Zubirán, Mexico City, Mexico; ^6^ Department of Clinical Immunology and Allergy, Royal Adelaide Hospital, Adelaide, SA, Australia; ^7^ Department of Immunopathology, SA Pathology, Adelaide, SA, Australia; ^8^ Division of Immunology, Transplants and Infectious Diseases, Università Vita-Salute San Raffaele, Milan, Italy; ^9^ Unit of Immunology, Rheumatology, Allergy and Rare Diseases, IRCCS Ospedale San Raffaele, European Reference Network (ERN) for Rare Immunodeficiency, Autoinflammatory and Autoimmune Diseases (RITA) Center, Milan, Italy; ^10^ Rheumatology and Clinical Immunology, Spedali Civili and Department of Clinical and Experimental Sciences, European Reference Network (ERN) for Rare Immunodeficiency, Autoinflammatory and Autoimmune Diseases (RITA) Center, University of Brescia, Brescia, Italy; ^11^ Clinical Unit of Autoinflammatory Diseases, Department of Autoimmune Diseases, Institut d'Investigacions Biomèdiques August Pi I Sunyer (IDIBAPS), Hospital Clínic of Barcelona, European Reference Network (ERN) for Rare Immunodeficiency, Autoinflammatory and Autoimmune Diseases (RITA) Center, University of Barcelona, Barcelona, Spain; ^12^ Rheumatology Unit, Department of Medicine, European Reference Network (ERN) for Rare Immunodeficiency, Autoinflammatory and Autoimmune Diseases (RITA) Center, University of Padua, Padua, Italy; ^13^ Department of Internal Medicine, Division of Rheumatology, Gazi University Hospital, Ankara, Türkiye; ^14^ Department of Medical Sciences and Public Health, University of Cagliari, Cagliari, Italy; ^15^ Rheumatology Unit, AOU Cagliari, Cagliari, Italy; ^16^ Department of Translational Medical Sciences, Section of Clinical Immunology, University of Naples Federico II, Naples, Italy; ^17^ Center for Basic and Clinical Immunology Research (CISI), WAO Center of Excellence, University of Naples Federico II, Naples, Italy; ^18^ Rheumatology Division, Faculdade de Medicina, Hospital das Clínicas, Universidade de São Paulo, São Paulo, Brazil; ^19^ Department of Precision and Regenerative Medicine and Ionian Area (DiMePRe-J) Policlinic Hospital, University of Bari, Bari, Italy; ^20^ Division of Rheumatology, Fondazione IRCCS Policlinico San Matteo, European Reference Network (ERN) for Rare Immunodeficiency, Autoinflammatory and Autoimmune Diseases (RITA) Center, Pavia, Italy; ^21^ Department of Internal Medicine and Therapeutics, Università di, Pavia, Italy; ^22^ Faculty of Medicine and Nursery, University of the Basque Country, UPV/EHU, Leioa, Biscay, Spain; ^23^ Autoimmune Diseases Unit, Biocruces Bizkaia Health Research Institute, Barakaldo, Biscay, Spain; ^24^ Rheumatology, Allergology and Clinical Immunology, Department of Systems Medicine, University of Rome Tor Vergata, Rome, Italy; ^25^ Molecular Medicine and Applied Biotechnology, University of Rome Tor Vergata, Rome, Italy; ^26^ Department of Biomedicine and Prevention, University of Rome Tor Vergata, Rome, Italy; ^27^ Department of Translational Hematology and Oncology Research, Taussig Cancer Institute, Cleveland Clinic, Cleveland, OH, United States; ^28^ Rheumatology Unit, Department of Medicine, University and Azienda Ospedaliera Universitaria Integrata of Verona, Verona, Italy; ^29^ Unit of Rheumatology, ASL VC Sant’ Andrea Hospital, Vercelli, Italy; ^30^ Department of Pediatrics, Giovanni XXIII Pediatric Hospital, University of Bari, Bari, Italy; ^31^ Clinica Medica “A. Murri”, Division of Internal Medicine, Department of Precision and Regenerative Medicine and Ionian Area (DiMePre-J), University of Bari Aldo Moro, Bari, Italy; ^32^ Rheumatology and Clinical Immunology Unit, Internal Medicine Department, Faculty of Medicine, Cairo University, Giza, Egypt; ^33^ Faculty of Medicine, Newgiza University, 6th of October City, Egypt; ^34^ Department of Pediatric Rheumatology, Faculty of Medicine, Hacettepe University, Ankara, Türkiye; ^35^ Department of Internal Medicine, Pneumonology, Allergology and Clinical Immunology, Central Clinical Hospital of the Ministry of National Defense, Military Institute of Medicine, National Research Institute, Warsaw, Poland; ^36^ Neuroscience Research Group (NEUROS), NeuroVitae Center, Escuela de Medicina y Ciencias de la Salud, Universidad del Rosario, Bogotá, Colombia; ^37^ Bioengineering and Biomedical Data Science Lab, Department of Medical Biotechnologies, University of Siena, Siena, Italy; ^38^ Ophthalmology Unit, Department of Medicine, Surgery and Neurosciences, University of Siena, Siena, Italy

**Keywords:** anti-TNF, anakinra, canakinumab, JAK inhibitors, tocilizumab, treatment

## Abstract

**Background:**

VEXAS syndrome, a recently identified systemic autoinflammatory disorder, poses new diagnostic and management challenges. Based on experience with other autoinflammatory diseases, anti-interleukin (IL)-1, anti-IL-6, anti-tumor necrosis factor (TNF) biotechnological agents, and Janus kinase inhibitors (JAKis) have been widely employed in VEXAS patients. The aim of this study is to evaluate the global effectiveness and safety of biotechnological agents and JAKis using data from the real-world context.

**Methods:**

Clinical, laboratory, and therapeutic data from VEXAS patients were obtained from the international AIDA Network VEXAS registry.

**Results:**

In total, 69 VEXAS patients were enrolled in the study. Among them, 12 patients (13 treatment courses) received IL-1 inhibitors, 12 patients (13 treatment courses) were administered anti-IL-6 agents, 8 patients (9 treatment courses) were treated with anti-TNF agents, and 16 patients (17 treatment courses) were treated with JAKis. A complete response was observed in 3 patients (23%) treated with anti-IL-1 agents, 2 patients (15%) receiving IL-6 inhibitors, 1 patient (11%) receiving TNF inhibitors, and 4 patients (23.5%) treated with JAKis. The mean prednisone (or equivalent) dosage significantly decreased during anti-IL-1 treatment (p = 0.01), while glucocorticoids changed during anti-IL-6, anti-TNF, and JAKi treatment in a non-significant fashion. A total of 21 patients experienced adverse events, 3 of which led to death (gut perforation, Legionnaires’ disease, and infectious pneumonia) while on JAKis; treatment withdrawal was required for 8 out of 21 patients.

**Conclusion:**

IL-1 and IL-6 inhibitors, along with JAKis, represent promising therapeutic options for VEXAS patients, albeit careful monitoring is mandatory to control disease activity and ensure safety.

## Highlights

• A notable percentage of patients benefit from at least partial disease control or even a complete response while on anti-interleukin (IL)-1 and anti-IL-6 agents and Janus kinase inhibitors (JAKis).
• As observed in this cohort, IL-1 antagonists could serve as effective glucocorticoid-sparing agents in VEXAS syndrome.
• Infections constitute approximately one third of adverse events in patients treated with biotechnological agents and JAKis, with injection site skin reactions particularly affecting IL-1 inhibitors and hematopoiesis being mostly affected by IL-6 inhibitors.
• The employment of JAKis represents a potential effective strategy, although their safety profile requires close monitoring, particularly regarding infectious adverse events.

## Introduction

Vacuoles, E1 enzyme, X-linked, autoinflammatory, somatic (VEXAS) syndrome, a recently identified systemic autoinflammatory disorder, poses new diagnostic and management challenges in the context of systemic inflammation. First described in 2020 by Beck et al. (2020), VEXAS syndrome is characterized by a complex spectrum of clinical manifestations that vary significantly including recurrent fever episodes, onco-hematological disorders, pulmonary involvement, vasculitis-related affections, various skin lesions, ocular/orbital manifestations, and thrombotic diathesis (Vitale et al., 2023). From a laboratory standpoint, patients show a remarkable increase in inflammatory indices and a frequent increase in the mean corpuscular volume (MCV). The presence of cytoplasmic vacuoles in hematopoietic precursors from bone marrow aspirates accounts for a further common characteristic in such patients (Obiorah et al., 2021). Unlike the classical monogenic autoinflammatory disorders, VEXAS syndrome is determined by somatic mutations of the *UBA1* gene, encoding for the first enzyme in the protein ubiquitination cascade, in the field of mosaicism; therefore, it typically arises in adulthood (Beck et al., 2020). The concomitant presence of other mutations, such as those involving the *DNMT3A* or *TET2* genes, known for their association with myelodysplastic syndromes or expanded hematopoietic clones, has been found in up to 24% of VEXAS cases (Georgin-Lavialle et al., 2022). The correct treatment approach for patients with VEXAS syndrome is still to be defined; to date, based on experience gained from other autoinflammatory diseases (Rigante et al., 2014), several treatment approaches have been attempted using conventional disease-modifying anti-rheumatic drugs (cDMARDs), biotechnological agents (bDMARDs), and small molecules, particularly Janus kinase (JAK) inhibitors (JAKis). Anti-interleukin (IL)-1, anti-IL-6, and anti-tumor necrosis factor (TNF) agents are the most widely used bDMARDs in VEXAS patients to date (Borie et al., 2023; Mascaro et al., 2023; Johansen et al., 2023; Oka et al., 2024; Vu et al., 2023). However, the current experience is based on case series and studies collecting data from a low number of patients (Heiblig et al., 2022; Bindoli et al., 2023). Therefore, this study was performed to ascertain the role of bDMARDs and JAKis in patients with VEXAS syndrome based on the real-world data collected in the international AutoInflammatory Disease Alliance (AIDA) Network registry dedicated to this disease (Vitale et al., 2022).

## Materials and methods

Patients with VEXAS syndrome were consecutively enrolled from November 2021 to March 2024 in the international AIDA Network registry dedicated to VEXAS syndrome (Vitale et al., 2022). Data collection was conducted prospectively, with laboratory and clinical information gathered starting from the time of enrollment into the AIDA registry. The follow-up period extended from the start of the symptoms to the last recorded assessment in the AIDA registry. The index date corresponded to the initiation of biotechnological agents and JAKis. Due to the lack of shared guidelines, patients’ treatment approaches were chosen by physicians according to their experience and on the basis of patient’s clinical features and disease activity.

The main objective of this study is to evaluate the global effectiveness of bDMARDs and JAKis employed in a relatively large cohort of patients diagnosed with VEXAS syndrome. Additional objectives of the study were to assess the safety profile in patients with VEXAS syndrome treated with bDMARDs and JAKis and understand how these treatment strategies are used in real life. Endpoints of the effectiveness were i) the frequency of a complete response, a partial response, and treatment failure and the persistence of symptoms at 3-month assessment after the start of therapies; ii) the decrease in daily prednisone (or equivalent) dosage between the start of treatment and the last follow-up visit while on treatment. The occurrence of adverse events and the treatment line in which bDMARDs were employed accounted for additional endpoints of the study to evaluate the safety profile.

Inclusion criteria required the presence of a pathogenic or likely pathogenic mutation in the *UBA1* gene, along with the onset of a systemic inflammatory condition not otherwise explained; the provision of signed informed consent for the utilization of clinical, laboratory, and genetic data within the AIDA network was also required (Vitale et al., 2022). The study was approved by the Ethics Committee of the Azienda Ospedaliero-Universitaria Senese, Siena, Italy, in June 2019 (Ref. N. 14951) as part of the AIDA Program. The study protocol adhered to the principles outlined in the Declaration of Helsinki.

Mutations in the *UBA1* gene and in genes associated with myelodysplastic syndromes or other onco-hematological disorders were detected through next-generation sequencing or Sanger testing, performed on peripheral blood or bone marrow samples obtained from patients. The presence of pathogenic or likely pathogenic mutations was an inclusion criterion for the primary study; information on the pathogenicity of the mutations was obtained from the Infevers database (https://infevers.umai-montpellier.fr/web/search.php?n=46) (Touitou et al., 2004; Milhavet et al., 2008; Van Gijn et al., 2018).

The disease duration was defined as the period between the onset of systemic inflammatory symptoms and the start of each specific bDMARD or JAKi. Skin involvement included neutrophilic dermatitis, vasculitic features, erythematous papules, erythema nodosum, and urticaria, which were previously reported to be associated with VEXAS syndrome. Arthritis was defined by the presence of at least one swollen joint or with signs of synovitis at ultrasound in at least one joint. Gastrointestinal involvement encompassed the presence of abdominal pain, diarrhea, and ulcerative lesions. Orbital/ocular involvement included episcleritis, uveitis, scleritis, blepharitis, periorbital edema, conjunctival chemosis, and eyelid edema, as previously reported. Cardiac involvement included acute myocardial infarction, myocarditis, and cardiac tamponade after the start of systemic inflammatory symptoms. Vessel involvement included stroke, critical limb-threatening ischemia, bowel infarction, pulmonary embolism, and deep vein thrombosis. Kidney involvement was defined as the presence of proteinuria, erythrocyturia with dysmorphic erythrocytes, and progressive renal disease leading to kidney insufficiency. Neurological involvement included minor or major cerebrovascular accidents, meningitis, and peripheral nervous system involvement, such as sensory neuropathy and multiple mononeuropathy (Vitale et al., 2023; Vitale et al., 2024). A concomitant disorder was an additional or related condition that occurred in the same individual in addition to VEXAS syndrome.

Regarding treatment outcomes, *complete response* was defined as the resolution of all disease-related clinical manifestations, with a decrease to normal values of all inflammatory laboratory parameters. *Partial response* was defined as the persistence of clinical manifestations with a remarkable decrease in their severity, as reported by patients and observed by physicians, with inflammatory laboratory parameters normalized or only slightly increased. *Failure* was defined as the persistence of VEXAS-associated clinical manifestations and/or no decrease in laboratory inflammatory markers. The term *adverse event* refers to any untoward medical occurrence observed after exposure to any treatment taken by the patients due for VEXAS syndrome, and it is not necessarily caused by the treatment.

The assessment of treatment responses was conducted for each patient at the last follow-up visit while on therapy, comparing observations at this final follow-up with those collected at the start of therapy. Laboratory assessment included the search for anemia, leukopenia, thrombocytosis, and thrombocytopenia at the time of disease onset. The reference ranges depended on the laboratory of the recruiting centers.

Regarding statistical computations, descriptive statistics included percentages, mean, standard deviation (SD), median and interquartile range (IQR), and frequency counts, as required. Qualitative data were analyzed using the Fisher exact test based on frequency counts and the expected frequencies. Quantitative data were analyzed using Student’s t-test or Mann–Whitney U test, according to data distribution assessed using the Shapiro–Wilk test. The association between treatment outcomes and any presence of myelodysplastic syndrome was assessed through multinomial regression. The significance level was set at 95% (*p*-value < 0.05); the *p*-value was two-tailed. Statistical analysis was performed using RStudio software, version 4.3.0.

## Results

In total, 69 patients diagnosed with VEXAS syndrome were enrolled. The mean age at disease onset was 66.4 ± (SD) 11.3 years, and the diagnosis occurred at 70.4 ± 10.9 years, with a median disease duration of 2.9 (IQR 4.4) years. Table 1 summarizes the demographic and clinical features of the enrolled patients. At enrollment in the international AIDA Network registry for VEXAS syndrome, 12 out of 69 patients (17.4%) were deceased. The causes of death were as follows: 2 cases succumbed to lung infection, while perforation of the intestine (nontraumatic), bacterial sepsis, Legionnaires’ disease, pulmonary heart disease, and acute pancreatitis were the causes of death in one patient each. However, data on 5 patients were missing.

**TABLE 1:** Clinical and laboratory features of VEXAS patients enrolled in this study. Details on oncological history are also provided.

Clinical and laboratory feature	
Sex (n female/male)	4/65
Age at enrollment, years (mean ± SD)	71.8 ± 8.0
Fever during disease exacerbations, n (%)	53 (76.8)
Skin involvement, n (%)	58 (84.1)
Orbital/ocular involvement, n (%)	33 (47.8)
Arthritis, n (%)	26 (37.7)
Chondral inflammation, n (%)	32 (46.4)
Gut involvement, n (%)	6 (8.7)
Neurological involvement, n (%)	12 (13.4)
Vessel involvement, n (%)	29 (42)
Cardiac involvement, n (%)	10 (14.5)
Lymphadenopathy, n (%)	15 (21.7)
Thoracic pain, n (%)	15 (21.7)
Kidney involvement, n (%)	3 (4.3)
Pleuritis, n (%)	8 (11.6)
Pericarditis, n (%)	1 (1.4)
Parenchymal lung involvement, n (%)	34 (49.3)
Orchitis, n (%)*	8 (12.3)
Epididymitis, n (%)*	6 (9.2)
Proteinuria, n (%)	5 (7.2)
Clinical history of neoplasms, n (%)	13 (18.8)
*Malignant neoplasm of prostate*	5 (38.5)
*Carcinoma in situ of skin*	2 (15.4)
*Malignant neoplasm of the lung*	1 (7.7)
*Carcinoma in situ of the bladder*	1 (7.7)
*Liposarcoma of the shoulder*	1 (7.7)
*Malignant neoplasm of the colon*	1 (7.7)
*Melanoma*	1 (7.7)
*Missing data*	1 (7.7)
Anemia, n (%)	63 (91.3)
*Macrocytic, n (%)*	51 (80.9)
*Microcytic, n (%)*	1 (1.6)
*Normocytic, n (%)*	11 (17.5)
Leukopenia, n (%)	34 (49.3)
*Lymphopenia, n (%)*	22 (64.7)
*Neutropenia, n (%)*	28 (82.4)
*Monocytopenia, n (%)*	4 (11.8)
Thrombocytosis, n (%)	2 (2.9)
Thrombocytopenia, n (%)	34 (49.3)
Paraproteinemia, n (%)	9 (13)
*MGUS, n (%)*	7 (77.8)
*Other, n (%)*	2 (22.2)
Concomitant hematological disorders, n (%)	34 (49.3)
*Myelodysplastic syndromes*	31 (91.3)
*Hodgkin’s lymphoma*	2 (15.4)
*Myelodysplastic/myeloproliferative neoplasms*	1 (2.9)
*B-lymphoblastic leukemia/lymphoma*	1 (2.9)
*Monoclonal gammopathy IgG kappa*	1 (2.9)

Abbreviations: MGUS, monoclonal gammopathy of uncertain significance; n, number.

Regarding the clinical picture, a total of 35 subjects (50.7%) with VEXAS syndrome had a defined rheumatologic/inflammatory syndrome associated with VEXAS. In particular, 21 patients (60%) had relapsing polychondritis, 8 patients (22.8%) presented with Sweet syndrome, 2 cases (5.7%) had been diagnosed with polyarteritis nodosa, 2 (5.7%) with spondyloarthritis, 2 (5.7%) with systemic lupus erythematosus, and 1 patient (2.8%) presented with polymyalgia rheumatica. Additionally, another subject (2.8%) suffered from giant cell arteritis. A malignant neoplasia was observed in 14 (20.3%) patients during their clinical history. Oncological details are reported in Table 1.

All patients presented a specific pathogenetic or likely pathogenetic mutation in the *UBA1* gene. Furthermore, 9 out of 38 subjects (23.7%) investigated for gene mutations associated with myelodysplastic syndromes or other onco-hematological disorders were found to carry such mutations (*DNMT3A* in 7 cases, *EZH2* in 1 case, and *TP53* in 1 case). Genetic testing was performed using the Sanger method in 25 patients (36.2%) and next-generation sequencing in 44 patients (63.8%); it was conducted on peripheral blood in 24 patients (34.8%) and bone marrow in 45 patients (65.2%). Specific mutations are detailed in Table 2. In total, 56 patients (81.2%) underwent bone marrow evaluation; the aspirate to search for vacuoles in hematopoietic precursors was reported in 25 patients (44.6%). Among them, 15 (60%) showed the presence of vacuoles in myeloid and erythroid precursors.

**TABLE 2:** Mutations identified in the *UBA1* gene and in other genes enhancing onco-hematological disorders among patients enrolled in this study.

Specific mutations in *UBA1* gene	n (%)
M41L (p.Met41Leu)	10 (14.5)
M41T (p.Met41Thr)	32 (46.4)
M41V (p.Met41Val)	18 (26.1)
p.Gly477Ala	3 (4.3)
p.Gly40_Lys43del	3 (4.3)
p.(Ser56Phe)	1 (1.5)
c.118-2A>G	2 (2.9)

Other mutated hematological genes^a^	n (%)
*DNMT3A*	7 (18.4)
*EZH2*	1 (2.6)
*TP53* ^b^	1 (2.6)

^a^ Calculated based on 38 patients who underwent specific genetic testing.

^b^ Notably, the TP53 mutation has been rarely observed in VEXAS patients. In this case, for patients suffering from myelodysplastic syndrome, the variant allele frequency (VAF) was low (1%), which could be associated with age-related clonal hematopoiesis.

A total of 59 subjects (85.5%) received glucocorticoids during their clinical history, and colchicine was administered to 21 patients (30.4%); a total of 35 subjects (50.7%) took cDMARDs, including methotrexate in 27 cases (77.1%), azathioprine in 10 subjects (28.6%), cyclosporine A in 8 patients (22.8%), hydroxychloroquine in 5 cases (14.3%), mycophenolate mofetil in 3 patients (8.6%), and leflunomide and cyclophosphamide in one patient (2.8%).

### bDMARDs’ effectiveness

**FIGURE 1:**
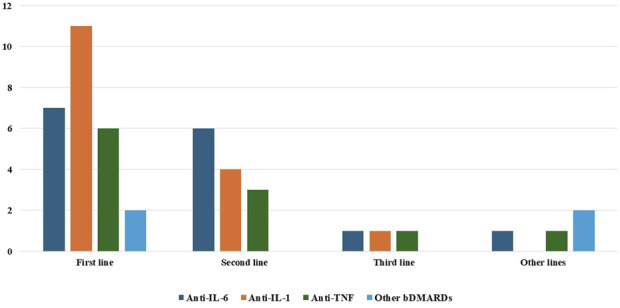
Distribution of use of the various biotechnological agents among the different biologic treatment lines. Abbreviations: bDMARDs, biologic disease-modifying anti-rheumatic drugs; IL, interleukin; TNF, tumor necrosis factor.

In total, 36 patients (52.2%) have been administered bDMARDs across 39 different treatment courses. The specific treatments used in this cohort are described in Table 3. Figure 1 shows the distribution of use of different bDMARDs at the various biological treatment lines.

**TABLE 3:** Frequency of patients treated with biotechnological and JAK inhibitors (in bold) and the number of treatment courses for each molecule (in italic) in this VEXAS cohort.

Biotechnological agent	n (%)
Anti-TNF	**8 (11.6)**
*Adalimumab*	*2*
*Etanercept*	*6*
*Infliximab*	*1*
Anti-IL-1	**12 (17.4)**
*Anakinra*	*11*
*Canakinumab*	*2*
Anti-IL-6	**12 (17.4)**
*Tocilizumab*	*12*
*Sarilumab*	*1*
Rituximab	**3 (4.3)**
Abatacept	**1 (1.5)**
JAK inhibitors	**15 (21.7)**
Ruxolitinib	**7**
Tofacitinib	**3**
Filgotinib	**3**
Baricitinib	**2**
Upadacitinib	**2**

**FIGURE 2:**
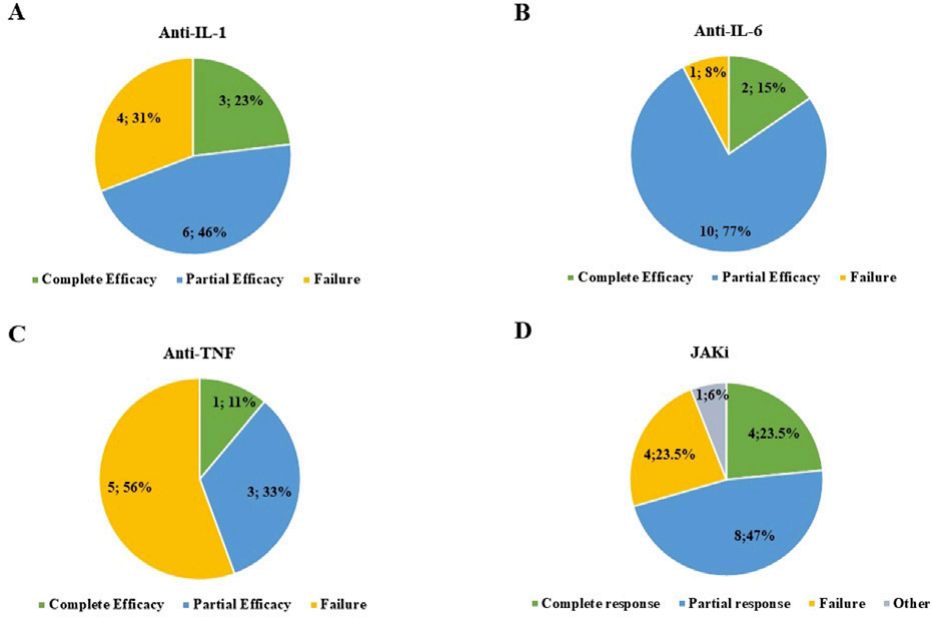
Frequency of patients experiencing complete response, partial response, and failure after treatment with interleukin (IL)-1 inhibitors **(A)**, IL-6 inhibitors **(B)**, anti-tumor necrosis factor (TNF) agents **(C)**, and Janus kinase (JAK) inhibitors **(D)**. In section D, “other” refers to one patient complicated with gut perforation while on treatment with baricitinib.

Anti-IL-1 agents were administered to 12 patients (17.4%) for a total of 13 treatment courses. The median follow-up period while on anti-IL-1 agents was 3 months (IQR = 6 and range 1–14). Anakinra was administered at a dosage of 100 mg/day to all patients except one, who was administered 100 mg every other day. One patient treated with canakinumab took 300 mg every 4 weeks; a second patient was treated with 150 mg every 4 weeks. Furthermore, 2 out of 11 (18.2%) patients treated with anakinra and 1 of the 2 patients treated with canakinumab benefited from a complete response; five (45.5%) patients treated with anakinra and one patient treated with canakinumab experienced a partial response; and four (36.4%) patients treated with anakinra did not benefit from anakinra introduction. Figure 2A provides the distribution of the outcome of IL-1 inhibition. Except for one patient treated with canakinumab, all patients undergoing IL-1 inhibition were treated with corticosteroids both at the start and last assessment. The mean prednisone (or equivalent) dosage was 20.2 ± 6.8 mg/day at the start of anti-IL-1 bDMARDs and 12.7 ± 7.0 mg/day at the last assessment (*p* = 0.01). One patient treated with anakinra was also administered cyclosporine 200 mg/day due to a lack of efficacy with the anti-IL-1 agent. The addition of cyclosporine did not improve the response. The odds ratio to experience partial efficacy rather than complete efficacy in patients with myelodysplastic syndrome compared to patients without myelodysplastic syndrome was 0.50 (95% CI: 0.02–11.8 and *p* = 0.66).

Anti-IL-6 was administered to 12 patients (17.4%) for a total of 13 treatment courses, followed up for a total of 4.5 months (IQR = 10.25, range 1–26). All patients except one were administered tocilizumab 162 mg/week subcutaneously; the last patient was treated with tocilizumab at a dosage of 8 mg/kg (480 mg) via intravenous infusion every 4 weeks. The patient treated with sarilumab took 150 mg every other week. A complete response was observed in two treatment courses (15.4%), while a partial response was reported in 10 treatment courses (including sarilumab), and two treatment courses were burdened by failure, as reported in Figure 2B. Two patients were concomitantly administered cyclosporine (100 mg/day in both cases); in one of them, the combination led to a complete response, which decreased after cyclosporine withdrawal. All patients undergoing IL-6 inhibition were treated with corticosteroids both at the start and last assessment. The median prednisone or equivalent dosage was 20.23 ± 12.67 mg/day at the start of the anti-IL-6 approach and 18.86 ± 11.8 mg/day at the last assessment (*p* = 0.92). One patient was concomitantly administered methotrexate 10 mg weekly, but this approach induced treatment discontinuation due to neutropenia.

The TNF inhibitors were used in eight patients (nine treatment courses) for a median follow-up period of 4 months (IQR = 8, range 2–44). Complete response was obtained in one treatment course (11.1%) with etanercept (50 mg/week). Partial response was obtained with three treatment courses (33.3%): adalimumab (40 mg weekly), infliximab (dosage not provided), and etanercept (50 mg/week). Failure was observed in five patients (55.5%), four of whom were treated with etanercept and one with adalimumab. Figure 2C showcases the clinical response to TNF inhibitors. Except for one patient treated with etanercept, all patients undergoing TNF inhibition were treated with corticosteroids both at the start and last assessment. The daily prednisone or equivalent dosage was 22.1 ± 11.9 mg/day at the start of anti-TNF treatment and 25.54 ± 14.14 mg/day at the last assessment (*p* = 0.77). One patient treated with etanercept was also treated with methotrexate at a dosage of 7.5 mg/week, later increased to 12.5 mg/week, with no final effectiveness. At multinomial regression analysis, the odds ratio to experience partial efficacy rather than complete efficacy in patients with myelodysplastic syndrome compared to patients without myelodysplastic syndrome was 0.99 (95% CI: 0.14–7.1 and *p* = 0.99); the odds ratio to experience failure rather than complete efficacy in patients with myelodysplastic syndrome compared to patients without myelodysplastic syndrome was 0.50 (95% CI: 0.05–5.5, p = 0.57).

All the patients treated with rituximab and abatacept experienced treatment failure.

In general, no statistically significant differences were observed in the frequency of a complete response among the different bDMARD classes (3/17 treatment courses with anti-IL-1 agents, 2/13 treatment courses with anti-IL-6 agents, and 1/9 treatment courses with anti-TNF; *p* = 1.00).

### JAK inhibitors’ effectiveness

In total, JAKis were administered to 15 patients (23.2%) for a total of 17 treatment courses. JAKis had been used after employing bDMARDs in 9 treatment courses (52.9%); in 4 treatment courses (23.5%), one bDMARD had been administered earlier, and in 4 cases (23.5%), at least two bDMARDs had been used before starting JAKis.

**FIGURE 3:**
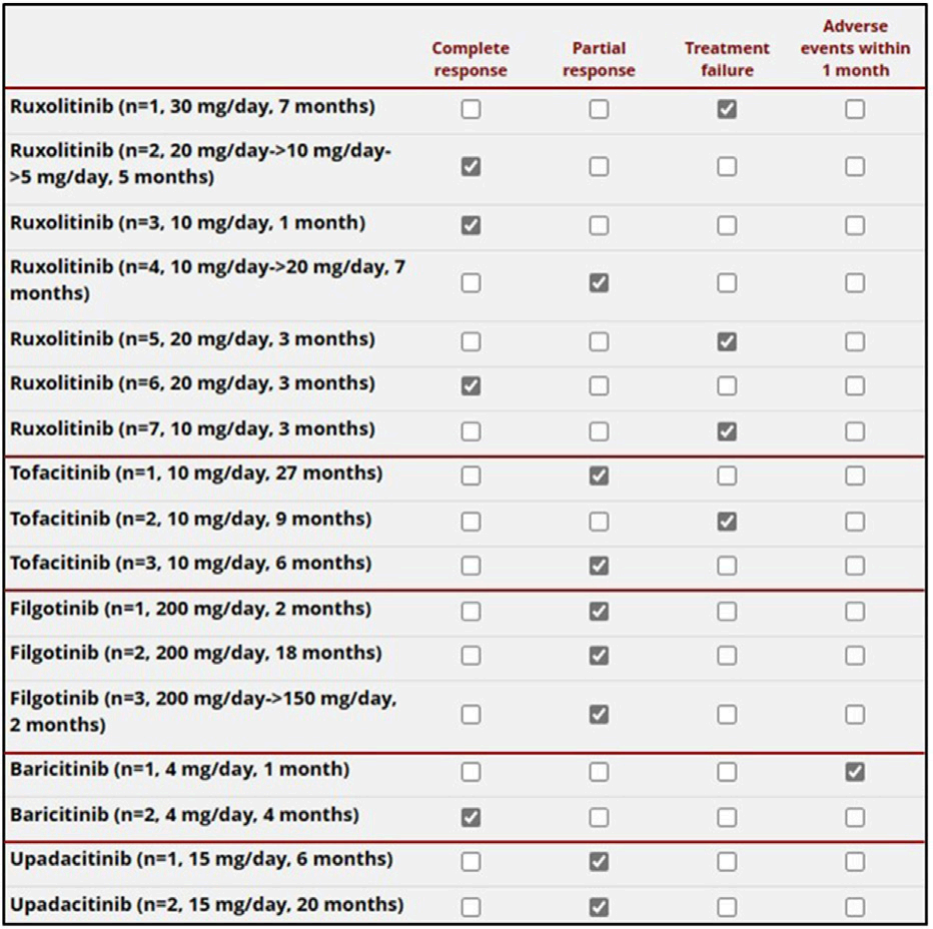
Details on the response to the JAK inhibitors administered to 16 patients. The daily posology and the total treatment duration are also provided in the first column on the left. One patient was first administered with upadacitinib and later with ruxolitinib. One patient treated with baricitinib was burdened by a severe adverse event (gut perforation) in the first month of treatment, prior to the establishment of global effectiveness.

The median follow-up while on JAKis was 6 months (IQR = 8, range 1–20). A complete response was observed in 4/17 patients (23.5%) treated with JAKis, while the frequency of partial response and failure was 8/17 (47.1%) and 4/17 (23.5%), respectively. The response was not assessed in one patient treated with baricitinib due to early discontinuation, as reported in Figure 2D. The specific details regarding the effectiveness of each specific JAKi are reported in Figure 3. All patients were concomitantly treated with glucocorticoids both at the start of JAKis and last assessment. In particular, the median prednisone (or equivalent) dosage was 17.5 mg/day (IQR = 21.25) at the start of JAKi and 11.25 mg/day (IQR = 16.25) at the last assessment (*p* = 0.16).

One patient treated with ruxolitinib was concomitantly treated with cyclosporine (100 mg/day), experiencing a complete response. None of the other patients concomitantly took cDMARDs.

**FIGURE 4:**
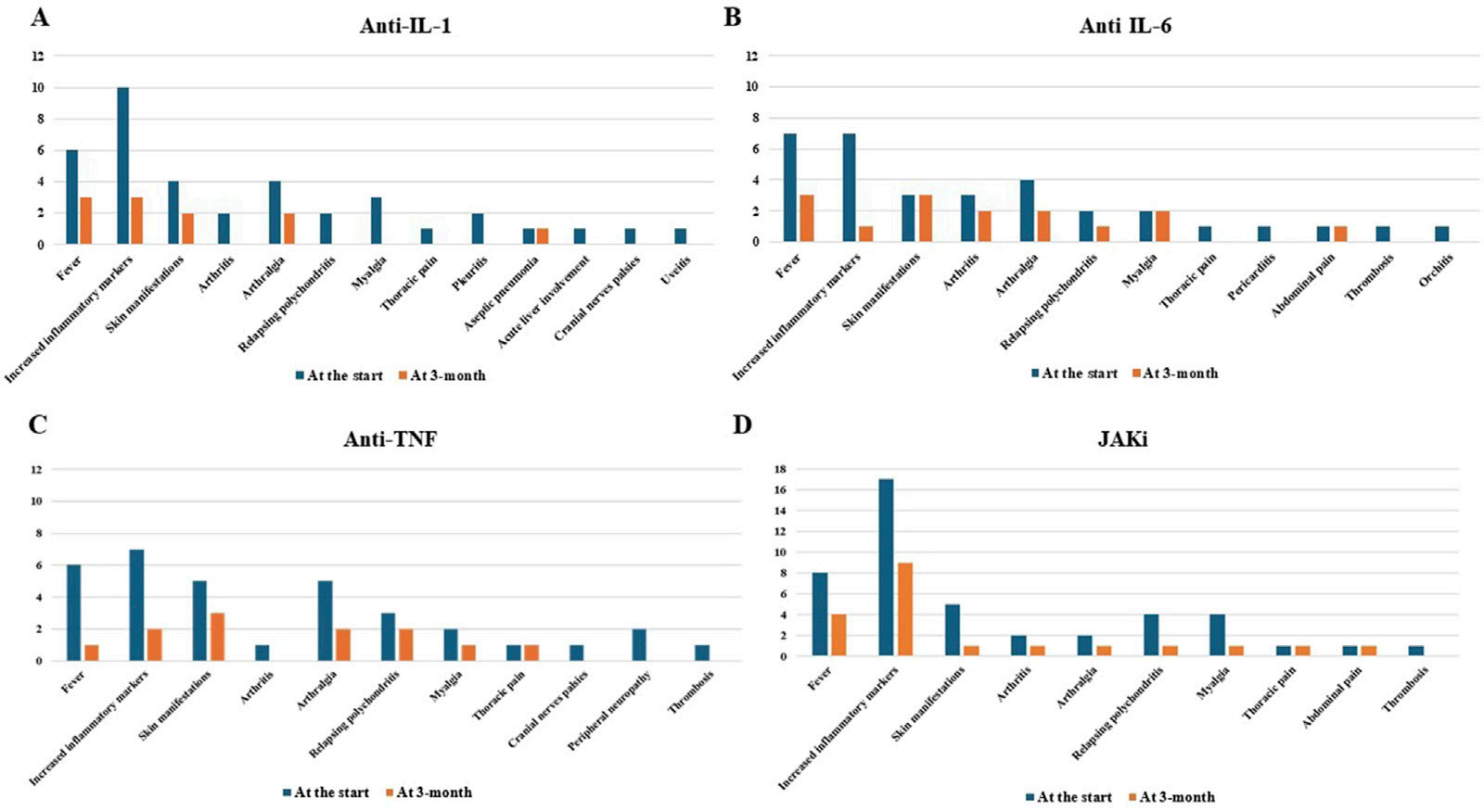
Clinical and laboratory manifestations observed at the start of anti-interleukin (IL)-1 **(A)**, anti-IL-6 **(B)** and anti-tumor necrosis factor (TNF) agents **(C)**, and Janus kinase (JAK) inhibitors **(D)** and that observed 3 months later.

Figure 4 provides information about the clinical and laboratory manifestations observed at the beginning of bDMARD and small molecule treatment and 3 months later.

According to multinomial regression analysis, the odds ratio to experience partial efficacy rather than complete efficacy in patients with myelodysplastic syndrome compared to patients without myelodysplastic syndrome was 10.02 (95% CI: 0.40–251.1, p = 0.16); the odds ratio to experience a failure rather than complete efficacy in patients with myelodysplastic syndrome compared to patients without myelodysplastic syndrome was 0.0003 (95% CI: 0–5.31 × 10^43^ and p = 0.88).

### Safety profile

A total of 21 patients experienced adverse events, four of which led to death and eight to treatment withdrawal, as detailed in Table 4. No adverse events were reported while on rituximab and abatacept. Table 5 provides information on peripheral blood cells at the start and last assessment while on bDMARDs and JAK inhibitors. Except for the number of white blood cells among patients treated with IL-6 inhibitors, no statistically significant differences could be observed between the start of treatment and the last assessment while on therapy. A trend toward statistical significance was observed in the percentage of lymphocytes between the start of treatment and the last follow-up visit, but this change led to the normalization of the value.

**TABLE 4:** Adverse events recorded during each of the treatment with biotechnological agents and JAK inhibitors.

Adverse event	n (%)
Anti-TNF	**4/8 (50)**
*Sepsis (etanercept)* ^a^	*1*
*Pneumonia (etanercept)*	*1*
*Upper respiratory tract infection (etanercept)*	*1*
*COVID-19 infection (etanercept)*	*1*
*Heart and kidney failure (infliximab)* ^a^	*1*
Anti-IL-1	**6/12 (50)**
*Skin reaction (anakinra and canakinumab)* ^a^	*2*
*Skin reaction (anakinra)*	*3*
*Neutropenia (anakinra)* ^a^	*1*
Anti-IL-6	**6/13 (46.2)**
*ARDS* ^b^	*1*
*Pneumonia (tocilizumab)* ^a^	*1*
*Pneumonia (tocilizumab)*	*1*
*Neutropenia (tocilizumab and sarilumab)* ^a^	*1*
*Neutropenia (tocilizumab)*	*1*
*Thrombocytopenia (tocilizumab)* ^a^	*1*
*Atopic reaction (tocilizumab)* ^a^	*1*
*Thrombocytopenia*	*1*
*Skin reaction*	*1*
JAK inhibitors	**6/16 (37.5)**
*Gut perforation (baricitinib)* ^b^	*1*
*Legionnaires’ disease (upadacitinib)* ^b^	*1*
*Pneumonia* ^b^	*1*
*Sepsis and DIC (baricitinib)* ^a^	*1*
*Neutropenia and thrombocytopenia (ruxolitinib)*	*1*
*Insomnia (tofacitinib)*	*1*

The number of patients is indicated in bold, while italic refers to the number of treatment courses.

Abbreviations: ARDS, acute respiratory distress syndrome; bDMARDs, biologic disease-modifying anti-rheumatic drugs; DIC, disseminated intravascular coagulation; IL, interleukin; TNF, tumor necrosis factor.

^a^ Leading to discontinuation.

^b^ Leading to death.

**TABLE 5:** Details obtained from peripheral blood cell count performed at the start and last assessment while on biotechnological agents and JAKis.

	Start of treatment	Last assessment	*p*-value
Anti-IL-1			
RBC, millions/mmc	3.7 ± 1.7	3.47 ± 0.69	0.87
Hb, g/dl	10.95 ± 1.8	11.42 ± 2.2	0.52
MCV, fl	105.4 ± 22.3	105.5 ± 14.8	0.99
WBC, n/mmc	4'668 ± 1′052	5'392 ± 2′445	0.53
Neutrophils (%)	65.35 ± 0.4	63.37 ± 31.4	0.92
Lymphocytes (%)	22.25 ± 2.47	29.30 ± 30.3	0.73
Platelets, n/mmc	185′500 ± 99013	123′500 ± 51427	0.21
Anti-IL-6			
RBC, millions/mmc	3.0 ± 0.84	3.2 ± 0.38	0.67
Hb, g/dl	10.3 ± 2.3	10.9 ± 1.4	0.58
MCV, fl	104.6 ± 9.1	105.1 ± 6.6	0.91
WBC, n/mmc	5′166 ± 2′228	2′953 ± 1′032	0.04
Neutrophils (%)	61 ± 19.3	51 ± 22.4	0.43
Lymphocytes (%)	45.6 ± 22.1	41.4 ± 21.5	0.74
Platelets, n/mmc	119′371 ± 39’770	120′125 ± 73’892	0.98
Anti-TNF			
RBC, millions/mmc	3.2 ± 0.7	3.1 ± 0.57	0.85
Hb, g/dl	10.25 ± 1	10.1 ± 1.3	0.9
MCV, fl	98 ± 17.5	102 ± 14.9	0.73
WBC, n/mmc	10′280 ± 9′111	10003 ± 7341	0.97
Neutrophils (%)	50 ± 19.5	56 ± 16.25	0.69
Lymphocytes (%)	42 ± 21.5	36 ± 16	0.71
Platelets, n/mmc	142′667 ± 24172	179′500 ± 106928	0.55
JAK inhibitor			
RBC, millions/mmc	3′307′778 ± 710′700	2′909′333 ± 1′220′919	0.41
Hb, g/dl	10.9 ± 1.6	10.4 ± 1.4	0.48
MCV, fl	98 ± 8.4	101.3 ± 10.9	0.78
WBC, n/mmc	6′061 ± 3′605	3′950 ± 2′212	0.12
Neutrophils (%)	64.3 ± 24.1	64.8 ± 15.7	0.96
Lymphocytes (%)	53.5 ± 25	32.1 ± 14.5	0.055
Platelets, n/mmc	186′000 ± 108′999	219′250 ± 155′626	0.57

Abbreviations: Hb, hemoglobin (grams per deciliter); IL, interleukin; MCV, mean corpuscular volume (femtoliter); RBC, red blood cells (millions per cubic millimeter); TNF, tumor necrosis factor; WBC, white blood cells (number per cubic millimeter).

## Discussion

During the last 3 years, numerous therapies have been attempted to manage the clinical and laboratory manifestations of VEXAS syndrome. bDMARDs and JAKis were among the first treatments to be proposed for these patients, largely based on the autoinflammatory nature of the disease and the concomitant on-label use of these drugs to treat the concomitant rheumatological comorbidities. These agents are generally used as alternatives to hypomethylating agents like azacitidine or decitabine (Mascaro et al., 2023; Comont et al., 2022; Mekinian et al., 2022). Allogeneic hematopoietic stem cell transplantation (AHSCT) is currently the only curative treatment for VEXAS and is a treatment option in severe cases unresponsive to other treatments (Mangaonkar et al., 2023). However, AHSCT is not devoid of complications and mortality, and other effective and safe therapeutic approaches should be identified for VEXAS patients (Diarra et al., 2022; Gurnari et al., 2024a). For these reasons, it is crucial to understand the true role that bDMARDs and JAKis may play in these patients, particularly in terms of effectiveness and safety profile, based on a sufficiently large number of patients.

In the present study, the percentage of patients achieving complete effectiveness with bDMARDs and JAKis seems relatively low. Nevertheless, a notable percentage of patients benefit from at least partial disease control or even a complete response. This is particularly true for IL-1 and IL-6 antagonists and JAKis. More in detail, roughly one-fourth of the patients treated with IL-1 inhibitors obtained a complete response, and two-thirds of the patients showed at least partial disease control. These findings are further corroborated by the parallel statistically significant glucocorticoid-sparing effect obtained with IL-1 antagonists after the start of the treatment. This is consistent with the findings of Borie et al., who described that 30.7% of VEXAS patients achieved clinical remission associated with a substantial tapering of glucocorticoid doses (Borie et al., 2023). Conversely, our outcomes seem better than those observed by Mascaro et al., who described a complete response in none of the 5 patients treated with anti-IL-1 agents and a partial response in 60% of cases (Mascaro et al., 2023). These discrepancies may depend on the heterogeneity of patients, especially considering the protean disease phenotype and the different pathogenic roles of *UBA1* mutations (Georgin-Lavialle et al., 2022). Our findings are more similar to those reported by van der Made et al., who described seven patients treated with anakinra, two of whom had a good response, one had a disease recurrence, and four discontinued treatments due to injection-site reactions (van der Made et al., 2022). This last issue is in line with what was observed in the present study regarding the high frequency of skin reactions in VEXAS patients treated with IL-1 inhibitors, especially anakinra. Injection site reactions were observed in 5/12 patients administered anakinra and canakinumab, two of whom necessitated treatment withdrawal.

The inhibition of IL-6 represents another promising treatment choice, with only 8% of cases showing no response and more than 90% of patients experiencing a positive result while on this approach. This supports a previous study on 15 VEXAS patients, 10 of whom showed a good response (Johansen et al., 2023). In the same perspective, Borie et al. described a clinical remission with a substantial glucocorticoid dose tapering in 15% of cases (Borie et al., 2023). A significant decrease in prednisone or its equivalent was not found in the present study; however, in contrast from what has been previously observed in the literature, all patients, except one, included in this study were treated with tocilizumab subcutaneously (Johansen et al., 2023; Kunishita et al., 2022; Tozaki et al., 2022). We wonder whether the intravenous administration of tocilizumab could have led to even better outcomes, particularly in terms of the glucocorticoid-sparing effect.

The TNF inhibitors used in our patients yielded complete effectiveness in only 11% of cases. A complete failure was described in more than half of the patients, making this therapeutic approach the one most frequently burdened by a lack of response. This is in line with what was observed by the FRENVEX study group, which observed a 0% response to TNF inhibitors (Hadjadj et al., 2024). In addition, the use of TNF inhibitors should be restricted in VEXAS patients due to the heightened risk of onco-hematological disorders, reported for both VEXAS syndrome and the use of anti-TNF agents (Gurnari et al., 2024b; Berghen et al., 2015; Wu et al., 2014). In this cohort, the use of TNF inhibitors had been started prior to the identification of *UBA1* gene mutations.

Regarding the use of JAKis, a complete response was reached in one-quarter of the patients, while an additional half of the cases obtained at least a partial response. In particular, the control of skin manifestations, which are a pivotal VEXAS feature, was evident in the present study (Figure 4D). According to the literature, the results obtained with JAKis substantially overlap with those observed by Mascaro et al. (2023) and Heiblig et al. (2022). In particular, based on 5 patients, Mascaro et al., reported a complete response in 20% of cases, a partial response in 40%, and no response in 40% (Mascaro et al., 2023). Heiblig et al. presented 30 VEXAS patients treated with JAKis, half of whom benefited from a clinical response as early as the first month of treatment; 20/30 patients showed a >50% reduction in CRP levels, including 11 with complete normalization of CRP. Eleven patients were still receiving treatment at the 6-month assessment, with 9 of them benefiting from a clinical response and 3 from complete CRP level control. A subgroup analysis disclosed significantly higher response rates in patients treated with ruxolitinib than in patients treated with other JAKis (Heiblig et al., 2022). However, despite similarities with previous studies, no direct comparisons may be carried out due to the different definitions of a response. According to Heiblig et al. (2022), our findings also seem to confirm a better outcome after ruxolitinib treatment as this JAKi achieves a complete response more frequently than other JAKis, as observed in Figure 3. Notably, the present study suggests a superior efficacy of JAK inhibitors that predominantly target both JAK1 and JAK2 (baricitinib and ruxolitinib) compared to JAK1-restricted inhibitors (filgotinib, tofacitinib, and upadacitinib). In this regard, as illustrated in Figure 3, a complete response was observed exclusively in patients treated with JAK1/2 inhibitors, whereas those receiving JAK1-restricted inhibitors predominantly achieved partial responses.

Despite the substantial decrease in the daily glucocorticoid dosage, with a median decrease of approximately 35%, the reduction in dose during JAKi administration did not reach statistical significance. This could be related to the greater severity of VEXAS syndrome among patients undergoing this treatment, as suggested by the high number of patients administered JAKi only after bDMARD discontinuation.

Establishing the real effectiveness of bDMARDs and JAKis cannot be separated from the corticosteroid-sparing effect. Complete effectiveness in terms of clinical and laboratory disease control may merely be achieved by maintaining relatively high dosages of prednisone or its equivalent. Therefore, a decrease in the daily prednisone or equivalent dosages is an essential endpoint when assessing the effectiveness of a treatment for VEXAS syndrome. In this regard, only IL-1 inhibitors could induce statistically significant glucocorticoid tapering, but in no cases was the median or mean prednisone dosage reduced below 10 mg/day. Moreover, despite the results described for anti-IL-6, the median daily dosage of glucocorticoids decreased from 20.23 mg/day at the start of the treatment to 18.86 mg/day at the last assessment. Therefore, regarding the results obtained with anti-IL-6 agents, which showed at least a partial response in nearly all patients, their limited effect as glucocorticoid-sparing agents should be considered, at least in the short term. In the same way, the complete discontinuation of glucocorticoids was quite uncommon for both bDMARDs and JAK inhibitors in this cohort, with only two patients discontinuing steroids: one treated with IL-1 inhibitors and one treated with etanercept. These findings suggest that the poor glucocorticoid-sparing effect of bDMARDs and JAK inhibitors may be due to the relatively short follow-up duration, which did not exceed 6 months, and also support the need for glucocorticoids in conjunction with these treatments, at least in the short term.

Patients with VEXAS syndrome are often elderly individuals, which enhances *per se* the risk for infections, while VEXAS syndrome seems to be associated with immunodeficiency (de Valence et al., 2024; Riescher et al., 2024). In this regard, infections constitute approximately one-third of adverse events reported in this study, with IL-1 inhibitors being the only class of bDMARDs not affected by infectious adverse events. Excluding the injection site skin reactions, which were frequent in patients treated with IL-1 inhibitors, anti-TNF agents accounted for the treatment approach more frequently burdened by a higher frequency of adverse events overall, especially infectious adverse events. Conversely, anti-IL-6 agents were more frequently responsible for hematopoietic impacts. Regarding JAKis, only 3 out of the 17 treatment courses were involved with infections, but three of the five adverse events reported in this class of drugs were represented by infectious complications. Of note, all the infectious adverse events leading to mortality occurred during JAKi treatment. This seems to comply with results reported by de Valence et al., who identified a higher susceptibility to infections among VEXAS patients treated with JAKis than among those treated with bDMARDs and azacitidine (de Valence et al., 2024). Notably, only two out of the five patients who experienced injection site reactions while on IL-1 inhibition required treatment withdrawal. According to literature data, patients with VEXAS syndrome frequently develop severe injection-site reactions after anakinra administration (Tan et al., 2024). However, in this cohort, anakinra was discontinued in only one out of four patients experiencing injection site reactions and in one patient treated with canakinumab. Both patients reported skin reactions after 6 months from the start of treatment. The remaining four patients experienced mild skin reactions shortly after the initiation of anakinra. As for other indications, these mild injection site reactions to anakinra were managed with several strategies, including warming the syringe to room temperature before injection, applying a cold pack to the injection site for 2–3 min before and immediately after the injection, using topical hydrocortisone or antihistamine cream, and alternating injection sites to prevent recall reactions (Kaiser et al., 2012).

During the last months, other interesting studies based on quite wide cohorts of patients have faced the topic of treatment for VEXAS syndrome. In particular, a study by the FRENVEX group, enrolling 110 patients treated with different bDMARDs and JAKis, identified a complete response and/or a partial response in 24% of patients treated with JAKis, 32% with IL-6 inhibitors, 9% with anti-IL-1, and 0% with TNFα blockers at the 3-month assessment. These percentages moved to 30% of patients treated with JAKis and 26% with IL-6 inhibitors at the 6-month follow-up (Hadjadj et al., 2024). These findings partly overlap with those observed in the AIDA cohort. However, notable differences emerge: the rates of patients achieving complete or partial responses to IL-1 inhibitors, IL-6 antagonists, and JAK inhibitors in the present study were significantly higher, reaching 69%, 92%, and 70.5%, respectively. This discrepancy likely stems from differing definitions of treatment responses. Specifically, the FRENVEX group applied more stringent criteria for treatment failure, requiring disease remission for both complete and partial responses. Additionally, their criterion of steroid sparing at 3 months could have induced too rapid tapering; our experience suggests that achieving steroid sparing could be a more gradual process (Hadjadj et al., 2024).

Interestingly, the concomitant presence of myelodysplastic syndrome—the most frequent hematologic manifestation of VEXAS syndrome—did not appear to play a role in determining therapeutic outcomes for either bDMARDs or JAK inhibitors. However, the wide 95% confidence intervals highlight the significant uncertainty associated with these results, relating to the need to increase the sample size. Although there is no statistical significance regarding the effect of myelodysplastic syndrome on the therapy response, the odds ratios seem to suggest a greater response to bDMARDs in patients with myelodysplastic syndrome and a partial response rather than a complete response in patients treated with JAK inhibitors. This should be the subject of future studies based on larger sample sizes.

The main limitations to this study are represented by the relatively small number of patients enrolled and the short follow-up duration. Furthermore, the multicenter study design (registry) implies heterogeneity in clinical practice, especially when dealing with disease lacking guidelines and recommendations. In addition, the completeness of the available data prevented us from understanding the role of the different therapies in avoiding blood transfusions. Furthermore, the real-life nature of the data prevented the identification of a fixed time point to evaluate the steroid-sparing effect of therapies from the start of each specific therapeutic approach. Patients were reviewed based on clinical needs rather than at pre-scheduled time points. Consequently, the steroid dose differences were evaluated over the entire study period. However, this study shows that the use of bDMARDs and JAKis appears to achieve at least partial clinical disease control in a remarkable number of VEXAS patients, disregarding any concomitant myelodysplastic syndrome. Due to the lack of more effective and safe therapy, employing bDMARDs and JAKis appears a valuable strategy for treating VEXAS syndrome at present. AHSCT, while representing a promising therapeutic opportunity, is burdened by non-trivial intrinsic mortality and should be deserved for particular cases (Gurnari et al., 2024a; Al-Hakim et al., 2022).

In conclusion, taking into account the limitations related to the sample sizes, which are not yet particularly large, the present study suggests that IL-1 inhibitors appear quite effective and safe for VEXAS patients, while IL-6 inhibition is useful, although more frequently leading to partial effectiveness. The employment of JAKis represents a potential effective strategy, especially with JAK1/2 inhibitors, although their safety profile warrants close monitoring. Overall, pending better therapies, anti-IL-1 and anti-IL-6 bDMARDs, along with JAKis, represent an opportunity for patients with VEXAS syndrome, albeit careful monitoring is essential to control disease activity and ensure treatment safety.

## Data Availability

The raw data supporting the conclusions of this article will be made available by the authors, without undue reservation.
